# Association of Chronic Myelogenous (Basophilic) Leukemia and the BCR/ABL Mutation in a Yucatan Barrow (*Sus scrofa domestica*)

**DOI:** 10.3389/fvets.2020.575199

**Published:** 2020-11-05

**Authors:** Catherine Takawira, Carmen B. Arsuaga-Zorrilla, Leslie Wilson, Takashi Taguchi, Marilyn A. Dietrich, Rhett W. Stout, Mandi J. Lopez

**Affiliations:** ^1^Department of Veterinary Clinical Sciences, School of Veterinary Medicine, Louisiana State University, Baton Rouge, LA, United States; ^2^Department of Pathobiological Sciences, School of Veterinary Medicine, Louisiana State University, Baton Rouge, LA, United States

**Keywords:** hematologic malignancy, Philadelphia chromosome, chronic myelogenous leukemia, flow cytometry, gene, pig, cancer, bone marrow

## Abstract

**Background:** Chronic myelogenous leukemia (CML) is a clonal proliferative disorder of the myeloid, megakaryocyte, and erythroid lineages. The onset and subsequent progression of CML is well-described in humans. There is comparably little information surrounding CML progression in veterinary species, including Yucatan miniature swine that are common for preclinical pharmaceutical and device testing. In humans, more than 90% of CML cases are associated with a chromosomal translocation that results in the Philadelphia gene (*BCR/ABL* mutation). In this report, the presence of the Philadelphia gene in a Yucatan burrow was confirmed in white blood cells collected prior to onset of clinical signs with primers designed from the human BCR/ABL sequence.

**Case Presentation:** A 24 month old, 70 kg, Yucatan barrow received a prefabricated bovine cortical bone xenograft following a unilateral zygomatic ostectomy for a preclinical study. Complete blood count and serum chemistries were performed prior to and 28, 53, 106, and 129 days after facial surgery. Fifty three days after surgery, a bone marrow biopsy was performed due to anorexia, severe basophilia, and mild anemia. A finding of a moderate increase in basophilic precursors in bone marrow cytology was followed by lymphocyte immunophenotyping via flow cytometry and RT-PCR amplification of the Philadelphia gene in white blood cell samples from the affected barrow and an unaffected barrow in the same treatment group. Bone marrow, lymph node, liver, spleen, lung, kidney, and adrenal gland lesions of mostly myeloblasts were identified after the affected barrow died 146 days after surgery. Flow cytometry confirmed lymphopenia and suggested basophilia, and RT-PCR established the presence of the BCR/ABL gene.

**Conclusions:** The information in this report confirms the presence of the BCR/ABL mutation and documents progression of chronic myelogenous (basophilic) leukemia from a chronic phase to a terminal blast crisis in an adult Yucatan barrow. The natural occurrence and progression of CML associated with the BCR/ABL mutation in miniature swine establishes potential for future porcine models of human CML. The information also establishes a genetic test to confirm porcine CML to prevent inadvertent attribution of clinical signs to treatment complications during preclinical testing.

## Introduction

Chronic myelogenous leukemia (CML) is a clonal proliferative disorder of the myeloid, megakaryocyte, and erythroid lineages (1, 2). It is reported in several species including swine, but there is limited description of clinical signs to warrant diagnostic testing for a premortem diagnosis (3–5). As such, the condition in swine is often identified post-mortem (6). The onset and subsequent progression of CML through an indolent “chronic phase” of mature granulocyte hyperproliferation to an aggressive and ultimately fatal “blast crisis” from clonal expansion of differentiation-arrested immature myeloblasts (blasts) is well-described in humans (5). There is comparably little information surrounding CML progression from early onset through the chronic phase and final blast crisis in veterinary species, including Yucatan miniature swine, a species central to preclinical pharmaceutical and device testing. Documentation of CML clinical progression, including standard diagnostic procedures, may help prevent inadvertent attribution of clinical signs to treatment complications during preclinical investigations (3, 7, 8).

More than 90% of CML cases in humans are associated with the Philadelphia gene (*Ph*+*)* (5, 9) while it is present in only 0.5–3% human cases of *de novo* acute myeloid leukemia (AML) (10). The gene results from fusion of the Abelson murine leukemia oncogene on chromosome 9 with the breakpoint cluster region of chromosome 22 (BCR-ABL translocation) (11). A BCR-ABL translocation between chromosomes 9 and 26, the “Raleigh” chromosome, was identified in several cases of canine CML (12–14). A potential association between chronic myelogenous (basophilic) leukemia and a Philadelphia gene sequence in Yucatan pigs is lacking in currently available literature (5, 9, 11, 12, 15, 16). Material in this novel report provides vital information that will contribute to accurate, pre-mortem diagnosis of *Ph*+ CML in outbred Yucatan swine.

## Case Description

A 24 month old, 70 kg, Yucatan barrow was acquired with 14 others from a closed herd in an Association for Assessment and Accreditation of Laboratory Animal Care International accredited, Public Health Service assured, pathogen-free breeding facility (Lonestar Laboratory Swine, Sioux Center, IA). Barrows were current on standard vaccinations and cared for following the *Guide for the Care and Use of Laboratory Animals* (17). They were housed with 4–5 others within pens (3.1 × 2.1 m) with concrete flooring covered by 2–3 inches of pine shavings (S & S Farms, Inc., Franklinton, LA). A pelleted miniature swine diet (Mazuri, Land O'Lakes, Inc., Saint Paul, MN) was provided twice daily, with water available *ad libitum*.

**Figure 1:**
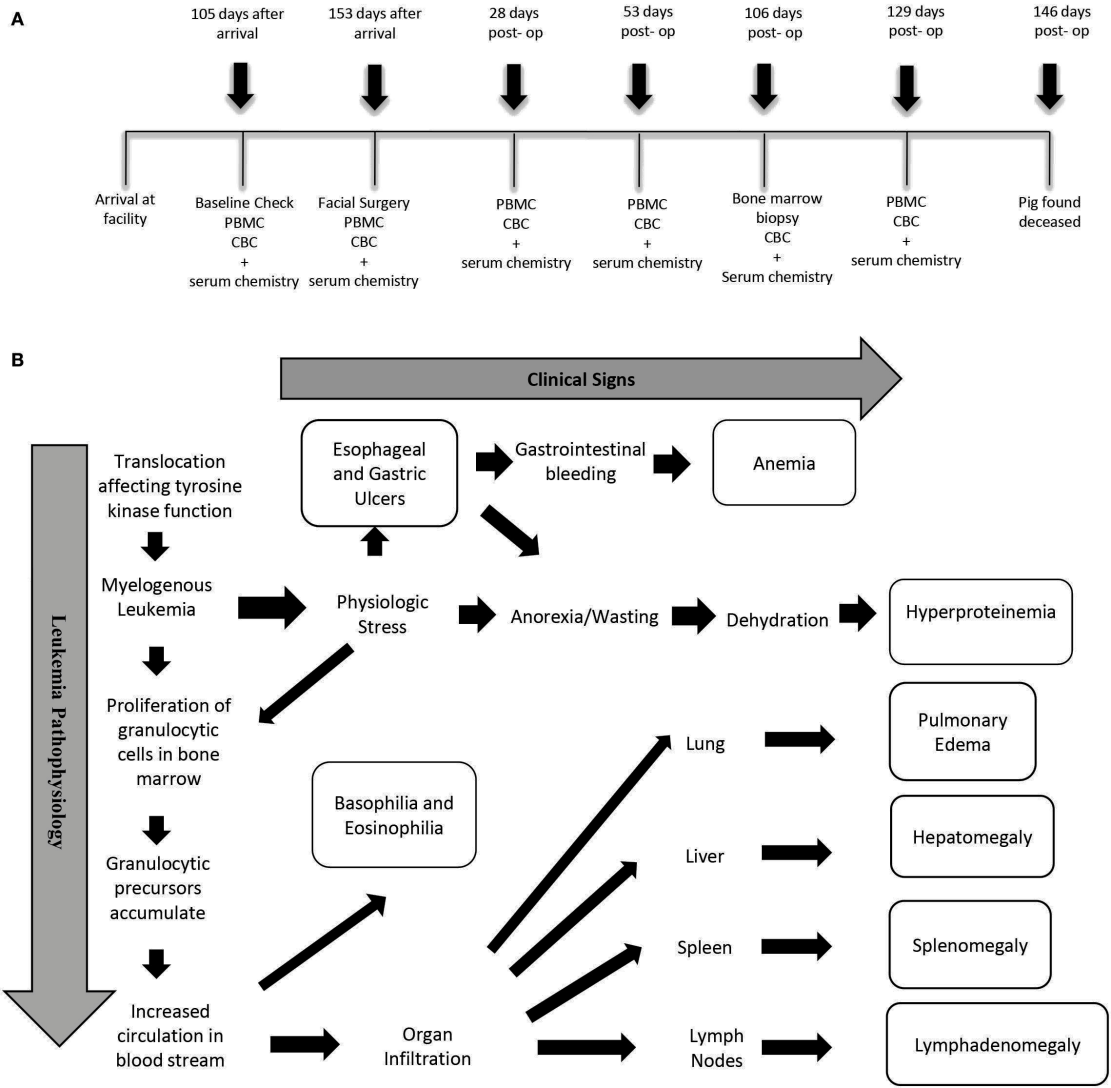
Timeline of diagnostic measures **(A)** and schematic of clinical signs and pathophysiology **(B)**.

After 4 weeks of acclimation, the barrows of this report were assigned to a treatment group that received a corticocancellous bone xenograft (bovine) following mandibular condylectomy as part of an Institutional Animal Care and Use Committee approved study. Blood was collected for complete blood count (CBC) and serum chemistries 105 and 153 days after arrival and 28, 53, 106, and 129 days after facial surgery (Figure 1). Aliquots of peripheral blood mononuclear cells (PBMCs) from blood samples were stored at −150°C. Baseline vital signs, CBC, and serum chemistries performed after acclimation were within normal limits (Table 1). Facial surgery was performed 153 days after arrival (Day 0). Due to chronic intermittent anorexia, persistent weight loss, and increased basophil percentages (Table 1) on CBC, a bone marrow biopsy was performed 106 days after facial surgery. The pig was found deceased 40 days later, and a complete necropsy, inclusive of histology, was performed. Lymphocyte immunophenotypes were quantified with flow cytometry using cryopreserved PBMCS (Supplementary Table 1). Philadelphia gene expression was determined using standard RT-PCR techniques with RNA extracted from PBMCs collected just prior to surgery.

**Table 1:** Complete blood cell count differentials, packed cell volume (PCV), and serum aspartate aminotransferase (AST) values of a Yucatan barrow with CML (affected) and a healthy barrow (healthy) over a period of about 6 months.

**Analytes**	**Reference intervals**	**Animal**	**105 Days after arrival**	**Facial surgery 153 days after arrival**	**28 Days Post-facial surgery**	**53 Days Post-facial surgery**	**106 Days Post-facial surgery**	**129 Days Post-facial surgery**
PCV (%)		Healthy	28	–	32	32	–	31
	RI:27-43	Affected	30	**24**	30	39	31	28
WBC (×103/ul)		Healthy	**10**	–	**8.5**	**9.1**	–	**9.4**
	RI:11–22	Affected	11.8	**25.5**	19.1	**23**	**26.2**	**24.1**
Abs SEG (×103/ul)		Healthy	5.5	–	2.9	4.8	–	3.8
	RI: 2–15	Affected	4.5	**19.1**	9.7	10.8	11.9	10.5
Abs LYMPH (×103/ul)		Healthy	4.2	–	4.7	4	–	5.3
	RI: 3.8–16.5	Affected	5.6	**3.7**	5.7	4.5	**3.3**	5.7
Abs MONO (×103/ul)		Healthy	0.1	–	0.4	0.4	–	0.3
	RI: 0–1	Affected	0.5	**1.5**	0	**1.4**	**1.3**	0.8
Abs EOS (×103/ul)		Healthy	0.2	–	0	0.1	–	0.1
	RI:0–1.5	Affected	0.2	0.1	0.2	0.3	0.5	0.7
Abs BASO (×103/ul)		Healthy	0	–	0	0	–	0
	RI: 0–0.5	Affected	**0.9**	**0.9**	**3.4**	**6**	0.5	**6.4**
AST (U/L)		Healthy	36		**56**	34	–	29
	RI:17–45	Affected	26	**16**	28	24	**109**	**275**

*Reference intervals (RI) are provided, and abnormal values are in bold font. Only significant chemistry abnormalities or changes are included in this table. Values are not available for the healthy barrow at all-time points. Abs, absolute; WBC, white blood cells; SEG, segmented neutrophils; LYMPH, lymphocytes; MONO, monocytes; EOS, eosinophils; BASO, basophils; AST, aspartate immunotransferase; PCV, packed cell volume*.

## Diagnostic Assessment

### Peripheral Blood Mononuclear Cell Isolation

Peripheral blood mononuclear cells were isolated, quantified, and cryopreserved using a previously described method (18). Briefly, the buffy coat was collected from whole blood in an EDTA tube (Becton Dickson Bioscience, Franklin Lakes, NJ) following centrifugation. It was mixed with an equal volume of fetal bovine serum (VWR Life Science, Radnor, PA), and the PBMCs were separated on a ficoll-paque gradient (Ficoll-Paque™ PLUS, Fisher Scientific, Logan, UT) by centrifugation at 400 × g for 30 min. The resulting PBMCs were washed with phosphate buffered saline (PBS) (Fisher Scientific) and resuspended in red cell lysis buffer for 3 min. An equal amount PBS (Fisher Scientific) was added and the mixture centrifuged at 400 × g for 10 min. Cells were resuspended in PBS (Fisher Scientific) and quantified with a hemocytometer. The PBMC suspension was centrifuged again at 400 × g for 10 min and cells resuspended in cryopreservation medium composed of 90% fetal bovine serum (Fisher Scientific) and 10% dimethyl sulfoxide (Fisher Scientific). Cell aliquots of 1 × 10^6^ cells/mL in cryopreservation tubes were slowly cooled to −80°C (CoolCell™ LX, BioCision, Tewksbury, MA) and subsequently transferred to and stored in liquid nitrogen (−150°C).

### Bone Marrow Harvest

While the affected barrow was under general anesthesia, a bone marrow biopsy was performed to determine the cause of a chronic basophilia. Following aseptic preparation, ~1.5 mL of bone marrow was aspirated from the iliac crest with a 10-gauge Jamshidi bone marrow needle attached to a 5 mL EDTA-coated syringe. The sample was used to make smear slides.

### Post-mortem Examination

The barrow was found deceased 146 days after facial surgery. A post-mortem examination was performed by a board certified veterinary pathologist. Samples of all major organs were fixed in 10% neutral buffered formalin, embedded in paraffin, and sections (5 μm) stained with hemotoxylin and eosin (H&E).

### Flow Cytometry

Flow cytometric analysis was performed on cryopreserved PBMCs. Briefly, cryovials were partially defrosted in a 37° water bath and then transferred to a 15 mL conical tube. A total of 7.5 mL of thawing media composed of 90% Roswell Park Memorial Institute medium (Sigma Aldrich) and 10% fetal bovine serum (Fisher Scientific) was added and followed by centrifugation at 400 × g for 10 min. Cells were resuspended in Roswell Park Memorial Institute medium and centrifuged as before. The cell pellet was resuspended in PBS (Fisher Scientific) and the viable cell number determined with a hemocytometer and trypan blue dye. Aliquots of 5 × 10^5^ viable cells suspended in 1 ml PBS were incubated with four labeled antibodies (Supplementary Table 1) in darkness at room temperature for 20 min, CD3e-fluorescein isothiocyanate (FITC, murine anti-porcine, #559582, Becton Dickson Bioscience), CD21-allophycocyanin (APC, murine anti-human, #559867, Becton Dickson Bioscience), CD8a-phycoerythrin (PE, murine anti-porcine, #559584, Becton Dickson Bioscience), and CD4a peridinin-chlorophyll-protein complex CY5.5 (PerCP-Cy™ 5.5, murine anti-porcine, #561474, Becton Dickson Bioscience). After incubation, samples were washed with PBS, fixed with 1% formalin and maintained at 4°C. Labeled cells were quantified with a flow cytometer (FACSCalibur, Becton Dickson Bioscience) utilizing 488 nm argon-ion and 635 nm red diode lasers. Samples were acquired with software (CellQuest^TM^ Pro, Becton Dickson Bioscience) on a computer workstation (Power Mac G5, Apple, Cupertino, CA) where dual fluorescence analyses are illustrated as dot plots. Unlabeled cells were evaluated to quantify autofluorescence. Cell debris was eliminated by gating on intact cells based on dot plots of forward scatter vs. side scatter. Percentages of cells expressing CD3, CD4a, CD8a, and CD21 subsets were determined simultaneously for each sample.

### Gene Expression

Total RNA was isolated from cryopreserved PBMCs (E.Z.N.A.® Micro Elute Total RNA kit, Omega BioTek, Norcross, GA) according to the manufacturer's directions. The RNA concentration was quantified spectrophotometrically (NanoDrop ND-1000; NanoDrop Technologies, Montchanin, DE, US), and RNA was reversed transcribed (Maxima First Strand cDNA synthesis kit, Fisher Scientific). Forward (5′-CATCATCCCTGCTTCTACC-3′) and reverse (5′-TGCTTCACCACCTTCTTG-3′) primers for porcine glutaraldehyde phosphate dehydrogenase (GAPDH) were designed from the porcine sequence (accession # XM_021091114.1). The human sequence for the Philadelphia gene (accession #AC026343.17) was used to design forward (ABL, 5′-AACTCGCAACAGTCCTTC-3′) and reverse (BCR, 5′-AGACATAAGCAGCAGTATCC-3′) primers. Both primers were designed with publicly available software (Primer-BLAST) and according to criteria established by the National Center for Biotechnology Information. Target gene levels were quantified with real-time quantitative PCR (SYBR® Green, Thermo Fisher Scientific; Chromo4^TM^, Bio-Rad Laboratories, Hercules, CA). Delta CT (ΔCt) values for ABL/BCR were determined relative to GAPDH as CT _reference_-CT _target_. The GAPDH and ABL/BCR gene amplicons were sequenced to confirm target sequence amplification (BigDye™ Terminator v3.1 Cycle Sequencing Kit, Applied Biosystems, Waltham, MA). The ABL/BCR amplicon was further localized on a 2% agarose electrophoresis gel with a 100 bp DNA ladder (New England Biolabs® Inc., Ipswich, MA). Amplicon sequences were compared to sequences in the National Center for Biotechnology Information database (CLC Sequence Viewer 8.0, QIAGEN, Hilden, Germany).

## Results

**Figure 2:**
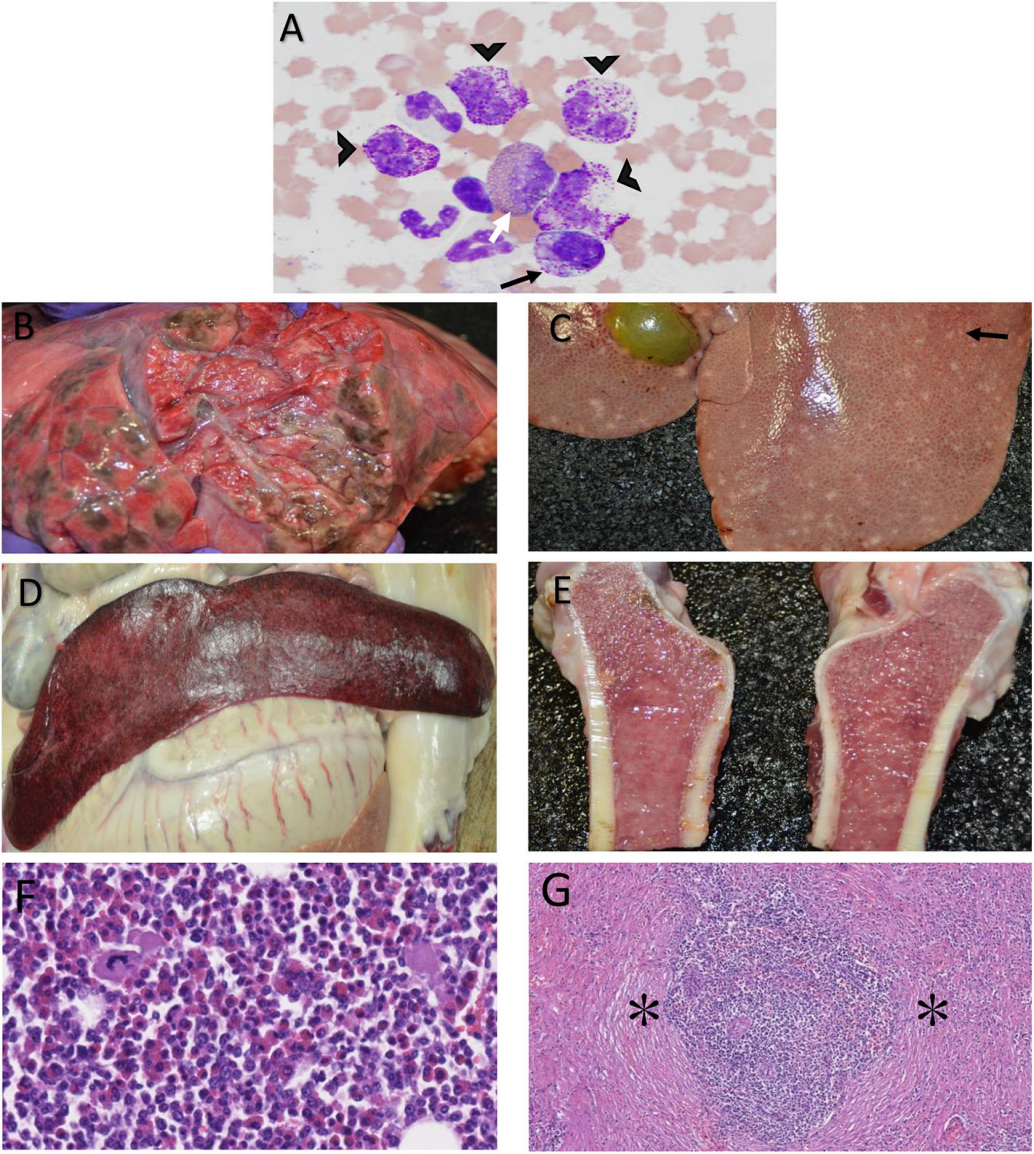
Photomicrographs of bone marrow cytology from a Yucatan barrow with chronic myelogenous leukemia depicting late stage basophilic precursors (**A**, black arrow heads), a midstage basophilic precursor (**A**, black arrow), and a mid-stage eosinophilic precursor (**A**, white arrow). Magnification = 40×, Stain = Wright Giemsa. Post-mortem photographs of the same barrow showing greenish-brown nodules in the lung parenchyma **(B)**, pale tan nodules (black arrow) throughout the liver **(C)**, a pale, moderately enlarged spleen **(D)**, and mottled pale tan to pink femur bone marrow, as can be seen with hyperplasia **(E)**. Bone marrow histology shows primarily myeloblasts mixed with fewer erythrocytic, leukocytic and megakaryocytic lines **(F)**. Histology of the spleen shows expansion of the parenchyma by stromal hyperplasia (asterisks, **G**). Magnification: F = 20×, G = 10×. Stain: H & E.

In general, the affected barrow had mild anemia (PCV 27–43%; reference interval 32–50%), mild leukocytosis (WBC 23–32.3 × 10^3^ μl; reference interval 11–22 × 10^3^ μl), mild lymphopenia (lymphocytes 3.3–3.7 × 10^3^ μl; reference interval 3.8–16.5 × 10^3^ μl), and mild to marked basophilia (basophils 0.9–6.4 × 10^3^ μl; reference interval 0–0.5 × 10^3^ μl) over the course of the disease. Notably, disease onset was characterized by a lymphopenia that fluctuated throughout the course of the disease and a more gradually progressive basophilia that peaked later than the lymphopenia. The barrow also had elevated aspartate aminotransferase (AST) 106 and 129 days after facial surgery (109 and 275 U/L, respectively; reference interval 17–45 U/L). Clinically insignificant fluctuations were seen in select CBC and chemistry parameters in the healthy barrow. Bone marrow cytology from the affected barrow showed a moderate increase in basophilic precursors with a slight left shift and mild increase in eosinophilic precursors (Figure 2A). A 500 cell differential count of nucleated cells consisted of 72% myeloid precursors (8% early-stage, 64% late-stage), 24% erythroid precursors (3% early-stage, 21% late-stage), and 4% megakaryocytes. Of the 64% late-stage myeloid precursors, 21% were late-stage basophilic precursors. The myeloid: erythroid ratio was mildly increased at 3 (reference interval 0.73–2.81). Both the myeloid and erythroid lineages displayed complete and synchronous maturation with no evidence of dysplasia. No overtly atypical cells or infectious organisms were observed. The presumptive diagnosis was moderate basophilic hyperplasia with mild eosinophilic hyperplasia.

There were lesions in multiple organs detected by gross necropsy. Multifocal, severe ulcers were present in the esophagus and gastric diverticulum with abundant gastrointestinal hemorrhage and marked enlargement of multiple gastric lymph nodes. The lungs were diffusely edematous, and there were multifocal to coalescing soft, greenish-brown nodules that extended into the parenchyma throughout the entire cranial and caudal lobes of the left lung (Figure 2B). The liver was diffusely pale and soft with multifocal, widespread, 1–3 mm diameter, pale tan nodules that extended into the parenchyma (Figure 2C). The spleen was pale and moderately enlarged (Figure 2D). The femur marrow was mottled pale tan to pink in color consistent with hyperplasia (Figure 2E).

On histologic assessment, there were non-encapsulated, poorly demarcated, infiltrative, moderately cellular sheets of myeloblasts expanding portal areas in the liver. There was marked femur marrow cellularity with ~90% similar myeloblasts admixed with fewer erythrocytic, lymphocytic, and megakaryocyte lines (Figure 2F). The lungs had variably-sized aggregates of lymphocytes with few plasma cells and mild to moderate edema. Vessels throughout the lung tissue were multifocally congested and contained high numbers of myeloblasts. Similar cells occasionally extended into the surrounding pulmonary interstitium. The gastric and mesenteric lymph node sinusoids contained histiocytes as well as myeloblasts. The splenic parenchyma was expanded by stromal cell hyperplasia and infiltration of myeloblasts (Figure 2G) that were also present in the kidney and adrenal glands. Chronic, moderate, diffuse enterocolitis and chronic, moderate, multifocal gastritis, both with lymphoplasmacytic and eosinophilic inflammation, was also present.

**Figure 3:**
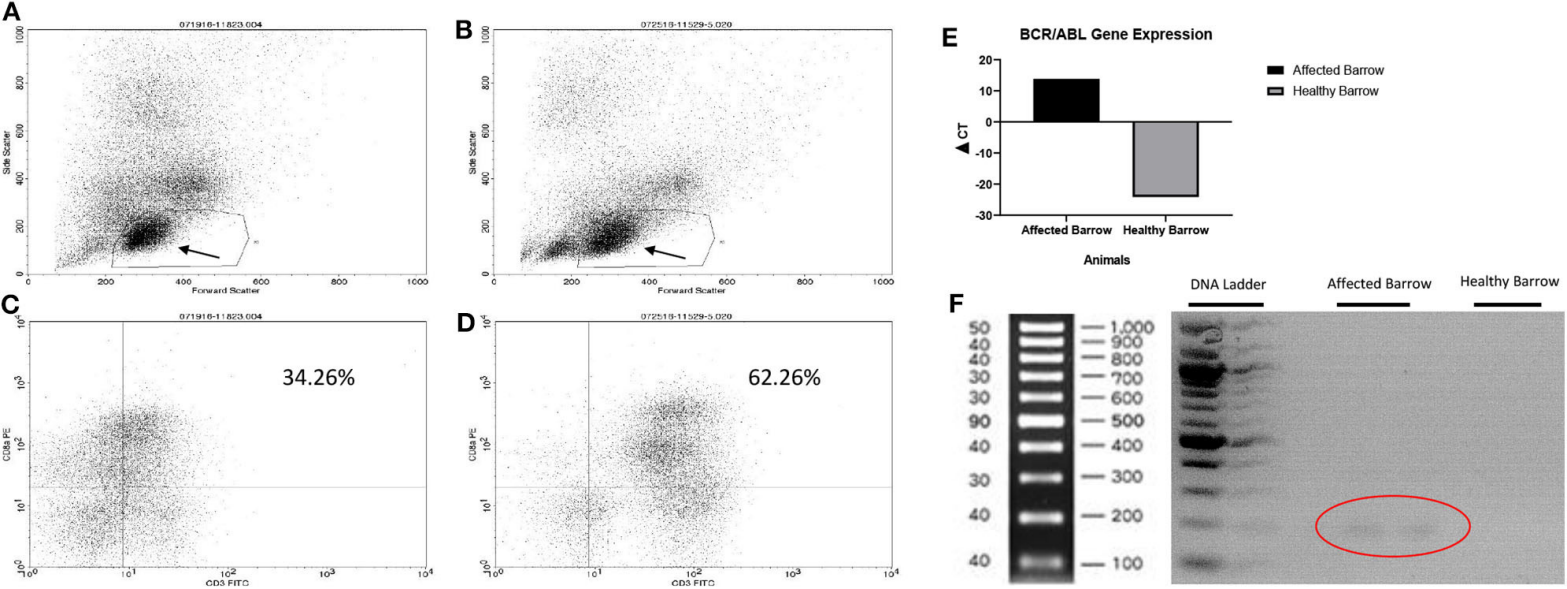
Flow cytometry plots demonstrating reduced CD3+/CD8+ cells in the barrow with leukemia **(A,C)** compared to an unaffected barrow **(B,D)**. Arrows demonstrate the lymphocyte region which may also contain degranulated basophils. Expression of the BCR/ABL fusion gene in Yucatan barrows with (affected barrow) and without (heathy barrow) chronic myelogenous leukemia **(E)**. Gel image demonstrating the BCR/ABL gene in the affected barrow (circle) **(F)**.

Based on flow cytometry, there were fewer lymphocytes in the affected (Figure 3A) compared to the healthy (Figure 3B) barrow. Specifically, there were fewer T lymphocytes (CD3+/8+) in the affected, 34.3% (Figure 3C), compared to the healthy, 62.3% (Figure 3D), barrow. All T lymphocyte subtypes, including CD3+/21– (natural killer & T cells), CD4+/8+ (double positive helper T cells), CD4–/8– (double negative regulatory T cells), and CD4+/8– (T helper cells), were lower in the affected vs. healthy barrow. The BCR/ABL fusion gene was expressed at detectable levels only in the affected barrow (Figures 3E,F). Multiple gene sequence alignment of PCR products predicted *Sus scrofa* GAPDH, transcript variant X2, mRNA (accession #XM_021091114), and *Sus scrofa* BCR, RhoGEF and GTPase activating protein, transcript variant X1, mRNA (accession #XM_021074056).

## Discussion and Conclusions

The information in this report addresses a gap in cytogenic investigations of hematologic malignancies within miniature swine in the veterinary scientific literature that lack the comprehensive clinical progression seen in this report for detection and diagnosis of naturally occurring porcine CML (16). Hematologic malignancies in miniature swine are frequently reported as incidental findings; most in depth reports are from line-bred or genetically engineered animals (Supplementary Table 2) (4, 5, 19). Reportedly, a BCR/ABL gene was identified *in vitro* within an immortal porcine cell line that exhibited spontaneous myeloid leukemic changes (4, 5). This report provides a porcine genetic marker associated with a fusion gene sequence using primers designed from the human forward ABL and reverse BCR sequences for the translocation. Use of the human sequences was necessary since there are currently no definitive porcine BCR or ABL sequences available (5, 20). The predicted region of fusion is not well-conserved due to high mutation rates. This and differences in human and porcine chromosome numbers are likely reasons that the porcine amplicon doesn't match that of the human BCR/ABL fusion (5, 16, 21–23). Nonetheless, the naturally occurring gene and CML signs and pathogenesis in the Yucatan barrow of this report are potentially applicable to research, production or pet miniature swine.

The barrow of this report displayed several signs and hematologic features that resemble the fatal blast crisis phase of CML in humans including an absolute basophilia, bone marrow hypercellularity, and splenomegaly (4, 24). Elevated AST may have been an early sign of hepatic involvement as it occurred at the same time that the peripheral basophilia peaked (5), though this cannot be confirmed without additional measures like sorbitol dehydrogenase and creatine kinase for liver disease and muscle damage, respectively, at the same time points. Further, acute myeloid leukemia cannot be entirely ruled out. Standard markers for leukemia like CD34 or acute myeloid leukemia such as CD123 were not tested, nor was special staining of cell granules performed. Flow cytometry analysis was used to confirm and refine the final CBC findings. Notably, there was no specific T cell lineage impacted more than another, confirming a general suppression of bone marrow lymphocyte generation associated with malignant basophil progenitors. As noted, the lymphocyte percentages on the affected barrow's plot did not contain normal levels of T and B lymphocytes. The lymphocyte region containing fluorescent CD3, CD4, CD8, and CD21 labels seemed to contain a cell type in addition to T and B lymphocytes. Based on the white cell differential, the cells may be basophils. Basophils lose their granules upon processing for flow cytometry analysis and ultimately appear in the lymphocyte region on a size vs. internal granularity dual parameter graphic plot (25, 26). Antibodies against porcine basophil antigens are necessary to confirm the presence of basophils in the flow cytometric dot plot.

Leukemia has one of the highest mortalities of any human cancer with CML accounting for 15–20% of all adult leukemias (27). Recently, myeloid leukemia and lymphoma occurred in a piglet that had severe combined immunodeficiency induced by transplant of unfractionated bone marrow from genetically engineered piglets with a haplotype 16 mutation of the Artemis gene (19). The information in the present report as well as the potential to induce leukemia via bone marrow transplantation (19) suggests that it may be possible to induce CML in Yucatan swine with genetically modified bone marrow cells. Such a model may provide important insight into pathogenic mechanisms and validation of novel therapeutic targets like tyrosine kinase inhibitor drugs that target the BCR-ABL protein (9) and microRNAs against genes associated with CML (27–29).

As mentioned previously, the presence of the Philadelphia gene is a hallmark of CML and only a rare subset of acute myeloid leukemias (AML) (30, 31). The barrow in this report appeared to display the standard stages of CML including a relatively insidious disease onset with a chronic phase in which symptoms waxed and waned followed by what might be considered a rapid or accelerated phase that progressed to a fatal blast crisis. Leukocytosis characterized by a mature neutrophilia is also a mechanism by which CML is often distinguished from AML which is more closely associated with blastocytosis (32, 33). One of the initial hematologic abnormalities in the affected barrow was a mild, non-specific increase in segmented neutrophils 153 days after arrival. Finally, in a CML blast crisis, a large percentage of neutrophils and a basophilia is typical, while in AML, a more normal differential without a basophilia occurs more frequently. Based on a recent report, documentation of an unexplained leukocytosis and the presence of the Philadelphia gene is sufficient for a CML diagnosis (34). The information in this report is limited by the fact that it is based on a single affected barrow. Future work is necessary to confirm the presence of the mutation in additional Yucatan miniature swine with clinical signs of CML. The results of this study confirm the presence of the BCR/ABL mutation, and clinical and post-mortem findings support a presumptive diagnosis of CML that progressed to a fatal blast crisis. Information in this report establishes a reliable mechanism to diagnose a genetically driven hematologic malignancy in miniature swine. The information may also contribute to diagnostic criteria and establish a premise for future swine models of CML.

## Data Availability Statement

The raw data supporting the conclusions of this article will be made available by the authors, without undue reservation.

## Ethics Statement

The animal study was reviewed and approved by the Louisiana State University Institutional Animal Care and Use Committee.

## Author Contributions

CT contributed to study design, performed assays, analyzed data, and wrote the manuscript. CA-Z performed the bone marrow biopsy and contributed to writing the manuscript. LW performed post-mortem and histological examinations. TT assisted with PCR performance and interpretation. MD performed flow cytometry and contributed to data interpretation. RS contributed to animal husbandry and writing the manuscript. ML conceived the study design, participated in or contributed to all diagnostic procedures and data analysis, and was a major contributor to manuscript preparation. All authors read and approved the final manuscript.

## Conflict of Interest

The authors declare that the research was conducted in the absence of any commercial or financial relationships that could be construed as a potential conflict of interest.

## Abbreviations

CML: Chronic myelogenous leukemia
BCR/ABL: Breakpoint cluster region/Abelson murine leukemia oncogene
Ph+: Philadelphia chromosome positive
RNA: Ribonucleic acid
APC: Allophycocyanin
PE: R-Phycoerythrin
PBMC: Peripheral blood mononuclear cell
PBS: Phosphate buffered saline
FBS: Fetal bovine serum
EDTA: Ethylenediaminetetraacetic acid
WBC: White blood cells
SEG: Segmented neutrophils
LYMPH: Lymphocytes
MONO: Monocytes
BASO: Basophils
PCV: Packed cell volume
AST: Aspartate immunotransferase.
