# Supplementary material for: Association of Chronic Myelogenous (Basophilic) Leukemia and the BCR/ABL Mutation in a Yucatan Barrow (Sus scrofa domestica)
Source: Front Vet Sci. 2020 Nov 5;7:575199. doi: 10.3389/fvets.2020.575199 (PMC7674400; doi:10.3389/fvets.2020.575199)
Supplement: Supplementary file 1 [file Table_1.pdf]

Antibody	Label	Marker	Host species	Target species	Manufacturer
CD4α	PerCP-Cy™ 5.5	T Lymphocyte	Murine	Porcine	BD Bioscience
CD3ε	FITC	T Lymphocyte	Murine	Porcine	BD Bioscience
CD8α	PE	T Lymphocyte & Natural Killer	Murine	Porcine	BD Bioscience
CD21	APC	B Cells	Murine	Anti-Human	BD Bioscience

Supplementary Table 1: Flow cytometric markers
