# Supplementary material for: Association of Chronic Myelogenous (Basophilic) Leukemia and the BCR/ABL Mutation in a Yucatan Barrow (Sus scrofa domestica)
Source: Front Vet Sci. 2020 Nov 5;7:575199. doi: 10.3389/fvets.2020.575199 (PMC7674400; doi:10.3389/fvets.2020.575199)
Supplement: Supplementary file 2 [file Table_2.pdf]

Swine Breed	Age/Sex	Clinicopathological Findings	Post Mortem Findings	Diagnosis
Miniature pitman-moore (Howard and Clarke, 1970)	66 - 88 months; male and female	Chronic leukopenia followed by eosinophilic leukemic hemogram, decreased ALP.	Varying degrees of marrow necrosis and fibrosis, neoplastic cell infiltration of liver, spleen, kidneys, lungs, gonads, adrenal, thyroid, and salivary glands, brain, heart, skeletal muscle and gastrointestinal tract	Myeloid metaplasia and myelogenous leukemia
Crossbred (Allsup et al., 1981)	3 days	Generalized yellow skin color	Icterus, enlarged lymph nodes, widespread petechiation, neoplastic infiltration of kidney, lymph nodes, spleen, and liver	Myeloid neoplasia
Landrace (Kashima et al., 1982)	36 months, sow	No clinical signs antemortem	Large numbers of eosinophils, promyelocytes and myelocytes as well as erythroblasts in blood smears, neoplastic cells in spleen, pancreas, digestive tract, kidneys post-mortem.	Eosinophilic myeloid leukemia
(Rafferty et al., 2007)	5 months, boar	Elevated heart rate, slow capillary refill time, cyanotic skin over ears and anemia	Creamy white bone marrow, petechial hemorrhages and nodules on kidneys and testis, large number of bone marrow lymphoblasts of B-cell origin	B-cell lymphoblastic leukemia
Landrace cross (Kadota et al., 1984)	30 months, sow	Reproductive disorders	Green myeloid lesions in bone marrow, spleen, lymph nodes, liver, ovaries, renal cortices, pancreas, endometrium, and mammary glands	Myeloblastic leukemia
Landrace, Landrace hybrid, Duroc (Kadota et al., 1987)	>24 months		Neoplastic involvement of bone marrow, spleen, liver, heart, adrenal glands, pancreas and lung neoplastic growth of eosinophilic promyelocytes	Myeloblastic leukemia and chronic myeloid leukemia
dd-haplotype (inbred for SLA-) miniature swine (Sipos et al., 2006)	48 months, sow	Herd infertility, weight loss progressing to recumbency, moderate enophthalmos, anemic conjunctiva and labial mucosa, hyperproteinemia, low-grade hypokalemia, hypomagnesemia, severe non-regenerative anemia, profound leukocytosis, moderate neutropenia, basophilia, peripheral blood myeloid cells co-expressing lymphoid markers	Severe lymphadenopathy with hepatomegaly, gray nodules in liver, kidneys and ovaries, splenic hemosiderosis and focal fibrosis, hepatic and lymph node myeloid white blood cell infiltration, renal and ovarian lymphoid infiltration, severe cystic hyperplasia of uterine glands	Undifferentiated leukemia
Inbred-MHC defined (SLA-d homozygous haplotype) (Duran-Struuck et al., 2015)Miniature swine	31-44 months, males and females	Lethargy, anorexia, weight loss, severe leukocytosis, anemia, peripheral blood mononuclear cells phenotypically dominated by myeloid phenotype with decreased lymphocytes	Organomegaly, lymphadenopathy, severe hepatosplenomegaly, pale bone marrow with 3:1 myeloid:erythroid ratio, tumor cells in lymph nodes and kidneys	Myelogenous leukemia

Supplementary Table 2: Representative porcine leukemia publications. ALP, Alkaline phosphatase; MHC, Major histocompatibility complex; SLA-d, Swine leukocyte antigen-d.
